# Diagnostic disclosure of Alzheimer's disease in Brazil: a national survey of specialized physicians

**DOI:** 10.1055/s-0043-1776316

**Published:** 2023-11-08

**Authors:** Vitor Santos de Souza, Sofia Brunchport Guazzelli, Leonardo Cardoso Cruz, Elisa de Paula França Resende, Leonardo Cruz de Souza, Maira Tonidandel Barbosa, Paulo Caramelli

**Affiliations:** 1 Universidade Federal de Minas Gerais, Hospital das Clínicas, Belo Horizonte MG, Brazil.; 2 Universidade Federal de Minas Gerais, Faculdade de Medicina, Belo Horizonte MG, Brazil.; 3 Universidade Federal de Minas Gerais, Faculdade de Medicina, Departamento de Clínica Médica, Belo Horizonte MG, Brazil.

**Keywords:** Alzheimer Disease, Diagnosis, Disclosure, Doença de Alzheimer, Diagnóstico, Revelação

## Abstract

**Background:** The diagnosis of Alzheimer's disease (AD) can bring financial and emotional consequences to patients and caregivers. Whether or not the diagnosis should be disclosed to patients is a matter of debate amongst physicians and can be influenced by culture and experience.

**Objective:** To investigate the current practice of physicians who attend and treat patients with dementia in Brazil regarding the disclosure of dementia diagnosis and compare the practice with what has been performed 15 years ago in the country.

**Methods:** Data were evaluated using an electronic questionnaire. The questions used to carry out this research were similar to the questions of the study carried out 15 years ago 9. The form was sent to the Brazilian Academy of Neurology, the Brazilian Association of Geriatrics and Gerontology, and the Brazilian Association of Psychiatry, which forwarded it to their members. Analyses were conducted through non-parametric statistical tests, with a post-hoc assessment.

**Results:** 397 physicians responded to the survey, of which 231 are neurologists, 124 geriatricians, 29 psychiatrists and 13 from other specialties. The mean age was 45.2 years. The majority (66.7%) of the physicians reveal the diagnosis of AD always or usually. The youngest group of neurologists were more likely to disclose the diagnosis than the oldest group with a significant difference between them. In comparison to the 2008 Brazilian study, the percentage of physicians who always or usually disclose the diagnosis has risen by 22%. On the other hand, 12.3% of the physicians rarely or never disclose the diagnosis, in comparison to 25,3% in 2008. The main reasons for not disclosing the diagnosis concern the patients' mental health.

**Conclusion:** Advances in dementia knowledge and biomarkers availability probably explain the increase in the rate of disclosure. The main challenge is to reconcile the autonomy of affected individuals, mental health issues after the diagnosis and the family member's opinion.

## Resumo

**Antecedentes:** O diagnóstico da doença de Alzheimer (DA) pode trazer consequências financeiras e emocionais para pacientes e cuidadores. Revelar ou não o diagnóstico aos pacientes é uma questão de debate entre os médicos e pode ser influenciada pela cultura e experiência.

**Objetivo:** Investigar a prática atual dos médicos que atendem e tratam pacientes com demência no Brasil quanto à revelação do diagnóstico de demência e comparar a prática com a qual era feita há 15 anos no país.

**Métodos:** Os dados foram avaliados por meio de um questionário eletrônico. As perguntas usadas para realização dessa pesquisa foram similares às perguntas do estudo realizado há 15 anos 9. O formulário foi enviado à Academia Brasileira de Neurologia, à Associação Brasileira de Geriatria e Gerontologia, e à Associação Brasileira de Psiquiatria, as quais o encaminharam aos seus membros. As análises foram realizadas por meio de testes estatísticos não paramétricos, com avaliação post-hoc.

**Resultados:** 397 médicos responderam à pesquisa, sendo 231 neurologistas, 124 geriatras, 29 psiquiatras e 13 de outras especialidades. A média de idade foi de 45,2 anos (standard deviation-SD = 11.6 years). A maioria (66,7%) dos médicos revela o diagnóstico de DA sempre ou habitualmente. O grupo mais jovem de neurologistas foi mais propenso a revelar o diagnóstico do que o mais velho, com diferença significativa entre eles. Em comparação com o estudo brasileiro de 2008, o percentual de médicos que sempre ou usualmente revelam o diagnóstico aumentou em 22%. Em contrapartida, 12,3% dos médicos raramente ou nunca o divulgam, em comparação a 25,3% em 2008. Os principais motivos para não o revelar dizem respeito à saúde mental dos pacientes.

**Conclusão:** Avanços no conhecimento da demência e disponibilidade de biomarcadores provavelmente explicam o aumento na taxa de divulgação. O principal desafio é conciliar a autonomia dos indivíduos afetados, problemas de saúde mental após o diagnóstico e opinião do familiar.

## INTRODUCTION

It is estimated that the number of people with Alzheimer's disease (pwAD) will double by 2050 worldwide, especially in low- and middle-income countries such as Brazil, 1 2 3 given the growing increase in life expectancy and the high prevalence of risk factors associated with the disease. 4 5 6

In this regard, the diagnosis of AD and its disclosure to affected people and their families, although challenging, are very important for the establishment of a pwAD-physician relationship, which is fundamental and known to improve trust, reliability and collaboration when facing the disease. The disclosure reinforces the autonomy of pwAD, in accordance with her/his right to self-determination, protecting her/his relationship with the physician and valuing their decisions. On the other hand, the paternalistic medical tradition recognized physicians as the only decision makers, giving them the right to withhold information from the affected individuals. This fact occurs mostly because truth telling imposes many obstacles, such as psychological distress, the physician's fear to take away hope and the fear to confess a bad prognosis. 7 This perspective is changing, being replaced by post-Flexner medical teaching models, which value patient autonomy and multidisciplinary health care. 8 Thus, the results of studies on the topic vary over time, depending on changes in society and its paradigms.

Fifteen years ago, Raicher et. al. asked 181 Brazilian physicians who often see patients with AD whether or not they disclose the AD diagnosis. 9 There were no significant differences between geriatricians, neurologists and psychiatrists regarding the frequency with which they informed patients of their AD diagnosis. Physicians' age was correlated significantly to AD disclosure, younger group more frequently reveals the diagnosis, while the older group more frequently rarely or never tells it. The results revealed that only 44.8% regularly inform the patient the diagnosis, which is influenced mainly by the patient's wish to be told. Despite this, 76.8% would like to know their own diagnosis if they were affected by AD.

Currently, the understanding about AD has evolved, and biomarkers became available with the possibility of a more precise and earlier diagnosis. However, disclosing the diagnosis is still a matter of debate, especially amongst different generations of doctors and family members. Therefore, the objective of this study was to investigate the current practice of physicians who attend and treat patients with dementia in Brazil regarding the disclosure of the diagnosis and factors that influence their behaviors.

## METHODS

After approval by the Ethics Committee of the Federal University of Minas Gerais, an electronic structured questionnaire ( Supplementary Material 1 - https://www.arquivosdeneuropsiquiatria.org/wp-content/uploads/2023/09/ANP-2023.0114-Supplementary-Material-1-e-2-.docx) in Google form format was sent to the aforementioned medical societies, which forwarded the form to their members. The questionnaire was open to responses for 85 days, from May to August 2022.

**Fig. 1:**
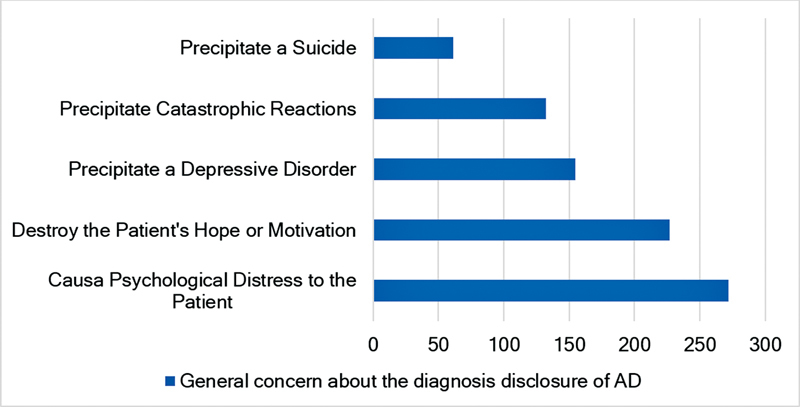
General concern about the diagnosis disclosure of AD.

The inclusion criteria of the research were: physicians who assist people with dementia. Based on the inclusion criteria, there was no need to create specific exclusion criteria.

The first part of the questionnaire had the informed consent form and demographic questions, which evaluated physicians' age, gender, graduation year, city of work, possible academic activity, how long they attend pwAD and how many patients they see per year.

The second part of the questionnaire evaluated factors associated to the pwAD which may influence the physician's decision to disclosure the diagnosis, namely: age; degree of confidence on the diagnosis of dementia or of a specific subtype of dementia; financial status; schooling; severity and stage of the dementia; pwAD desire to know the diagnosis; the family opinion on the diagnostic disclosure; comorbidities; personality of the pwAD and the possibility that the diagnostic tests are inconclusive. Also, factors associated to the diagnostic disclosure itself that influence the physician's decision were also evaluated, such as the potential of causing psychological distress to the pwAD; to precipitate catastrophic reactions, a depressive disorder; suicide; or the possibility of the disclosure taking away hope and motivation.

The data were computed on Excel and analyzed on SPSS software. Non-parametric Chi-square test was used. Considering the large sample size, to evaluate the statistical differences found, the alpha value was corrected and a post-hoc assessment was performed.

It is worth remembering that a similar study was carried out just over 15 years ago. 9 We replicated the same questionnaire to also check if there were any changes.

## RESULTS

Overall, 397 physicians who often attend pwAD responded to the survey. All questionnaires were correctly completed. Exactly 51.6% of the respondents were male, with a mean age of 45.2 years (standard deviation-SD = 11.6 years). All regions of the country were represented, with a greater number of neurologists and geriatricians, mainly from the Southeast and Northeast regions ( Supplementary Material 2 - https://www.arquivosdeneuropsiquiatria.org/wp-content/uploads/2023/09/ANP-2023.0114-Supplementary-Material-1-e-2-.docx). Of the 26 states in the country, responses were obtained from 21 states and the Federal District. Precisely 67.5% of physicians had some university activity by the time of the survey.

**Fig. 2:**
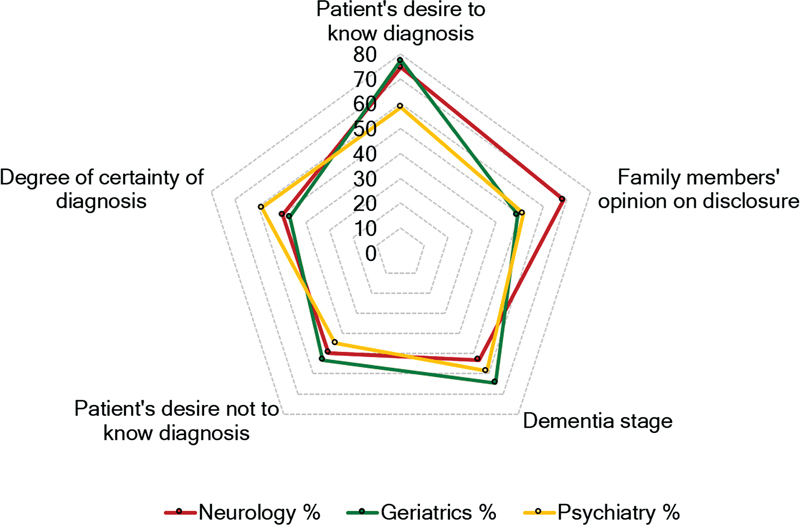
Main characteristics that influence the decision to disclose or not the diagnosis of AD according to the physician's medical specialty.

Table 1 depicts the demographic data from all respondents and the frequency of AD diagnosis disclosure.

**Table 1:** Demographic informations

		Neurologists N 231 (58.2%)	Geriatricians N 124 (31.2%)	Psychiatrists N 29 (7.3%)	Others N 13 (3.3%)	Total N 397 (100%)
**Age group-N (%)**	25-45 years	136 (58.9%)	66 (53.2%)	20 (69.0%)	10 (76.9%)	232 (58.4%)
	46-85 years	95 (41.1%)	58 (46.8%)	9 (31.0%)	3 (23.1%)	165 (41.6%)
**Gender-N (%)**	Male	136 (58.9%)	48 (38.7%)	15 (51.7%)	7 (53.8%)	206 (51.9%)
	Female	95 (41.1%)	76 (61.3%)	14 (48.3%)	6 (46.2%)	191 (48.1%)
**University Activity-N (%)**	Yes	168 (72.7%)	71 (57.3%)	21 (72.4%)	8 (61.5%)	268 (67.5%)
	No	63 (27.3%)	53 (42.7%)	8 (27.6%)	5 (38.5%)	129 (32.5%)
**Diagnostic disclosure-N (%)**	Always or usually	156 (67.5%)	77 (62.1%)	22 (75.9%)	10 (76.9%)	265 (66.7%)
	Never or rarely	26 (11,3%)	17 (13.7%)	5 (17.2%)	1 (7.7%)	49 (12.3%)

Tables 2 and 3 display the diagnostic disclosure rates of geriatricians and neurologists, given that these specialties included 355 physicians (31.2% and 58.2% of participants, respectively). Most physicians reveal the diagnosis of AD always or usually, mainly within the youngest group (25-45 years-old) group that disclose the diagnosis more frequently than the oldest group of physicians ( Table 2 ). The proportion of physicians within the 25-45 years old range who never or rarely disclose the diagnosis is significantly lower than the 46-85 years group.

**Table 2:** displays the diagnostic disclosure rates of geriatricians and neurologists, given that these specialties included 355 physicians (31.2% and 58.2% of participants, respectively)

	Always or usually-N (%) ^a^	Sometimes-N (%)	Never or rarely-N (%) ^b^	Total-N (%)
**25-45 years**	150 (74.3%)	36 (17.8%)	16 (7.9%)	202 (100%)
**46-85 years**	83 (54.2%)	43 (28.1%)	27 (17.6%)	153 (100%)
**Total**	233 (65.6%)	79 (22.3%)	43 (12.1%)	355 (100%)

Notes: Chi-squared X ^2^ (2) = 16,246; alpha = 0.0083 ^a^ ;p = 0.0001 ^b^ ;p = 0.0051; Cramer's V 21%.

**Table 3:** Displays the diagnostic disclosure rates of geriatricians and neurologists, given that these specialties included 355 physicians (31.2% and 58.2% of participants, respectively)

		Always or usually	Sometimes	Never or rarely	Total
**Geriatricians-N (%)**	**25-45 years**	45 (68.2%)	15 (22.7%)	6 (9.1%)	66 (100%)
	**46-85 years**	32 (55.2%)	15 (25.9%)	11 (19.0%)	58 (100%)
	**Total**	77 (62.1%)	30 (24.2%)	17 (13.7%)	124 (100%)
**Neurologists-N (%)**	**25-45 years**	105 (77.2%)*	21 (15.4%)	10 (7.4%)	136 (100%)
	**46-85 years**	51 (53.7%)*	28 (29.5%)	16 (16.8%)	95 (100%)
	**Total**	156 (67.5%)	49 (21.2%)	26 (11.3%)	231 (100%)

Notes: alpha = 0.0083; *p < 0.0001; Cramer's V 25%.

Amongst geriatricians, there was no difference between the two age groups in disclosing the AD diagnosis ( Table 3 ). However, amongst neurologists, the youngest group (25-45 years old) significantly reveal more the diagnosis always or usually than the older group (46-85 years old).

Issues generally causing concern surrounding the disclosure of the diagnosis to pwAD are summarized in Figure 1 . The geriatricians', neurologists' and psychiatrists' main concerns are the possibility of causing psychological distress to the affected individual in 70.2%, 68.4% and 62.1%, respectively, and to destroy the person's hope or motivation in 49.2%, 61.9% and 51.7%, respectively.

The main factor influencing whether or not to disclose the AD diagnosis to the pwAD was the wish to be told (74.3%), as seen in Figure 2 . For neurologists, the decision to disclose has been taken based on the pwAD desire to know the diagnosis and the family's opinion about disclosure. Geriatricians mainly consider the pwAD desire and the stage of dementia, while psychiatrists were more influenced by the degree of diagnostic certainty.

**Fig. 3:**
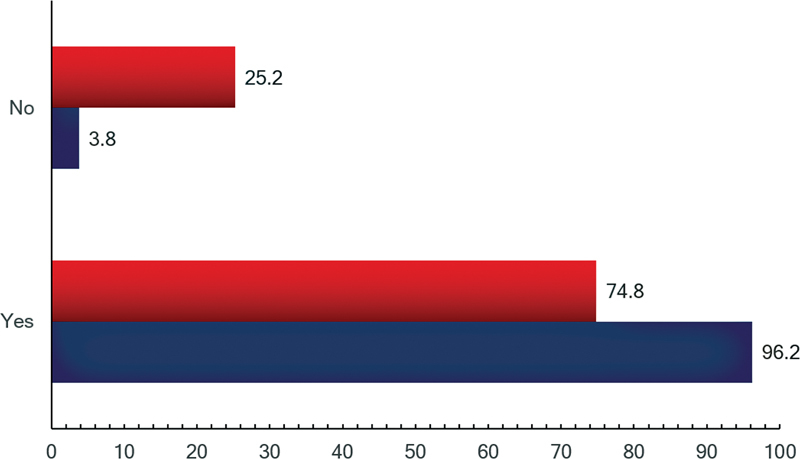
Opinion about the Alzheimer's disease diagnosis disclosure.

When physicians were asked about their opinion on the pwAD desire to know their diagnosis, 75% believe that they do want to know and 25% believe they do not. Regardless of the frequency with which neurologists and geriatricians reveal the diagnosis of AD, they believe pwAD want to know their own diagnosis (p = .000; Cramer's V 43%). On the other hand, if these professionals were diagnosed with AD, 96% would like to know their own diagnosis, as exemplified in Figure 3 . The same p-value was found for this variable, except for the professionals who only sometimes reveal the diagnosis (p = 0.368).

The reasons why professionals would like to know their own diagnosis were related to the intention to prepare for the future (49.4%) and to deal in advance with issues associated with the management of their assets (25.2%).

**Fig. 4:**
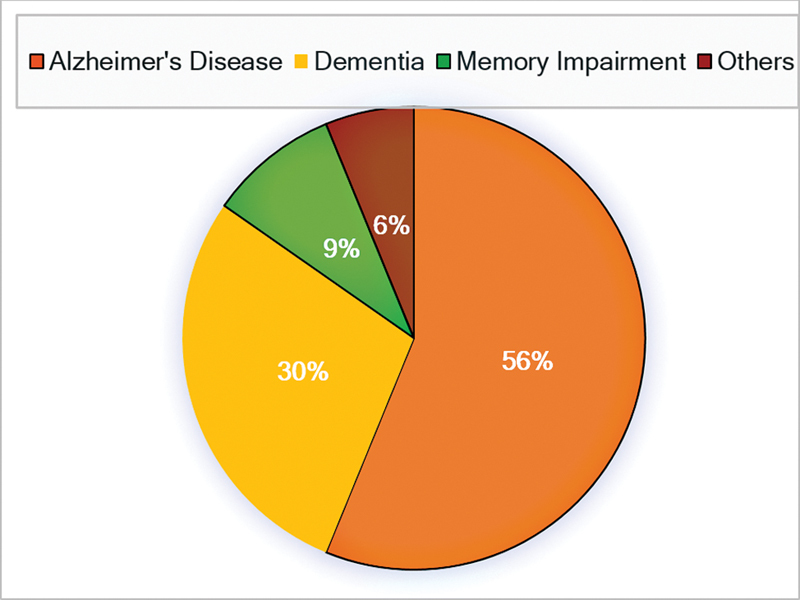
Nomenclature used by physicians to diagnose patient.

The nomenclature in referring to the disease used by the physicians was also examined: 84.7% of the respondents always used clear terminology such as AD or dementia, and the rest used a variety of terms including “memory impairment,” “forgetfulness,” “senility” or “sclerosis”, as shown in Figure 4 .

For physicians who assisted pwAD for more than 10 years (n = 259), 33.2% changed their conduct, now revealing the diagnosis. The main reason for the change in the conduct of physicians was that the diagnosis became more accurate with the development of new methods, such as biomarkers (24.1%); however, many participants could not specify the main reason for the change in their behavior (38.2%). Meanwhile, 17.8% still do not reveal the diagnosis and 49.0% continue to reveal the diagnosis. The main cause of persistence with the approach of not revealing the diagnosis was fearing of the pwAD's psychological reaction.

In relation to the discovery and the use of biomarkers in the diagnosis, 46.1% of physicians had their conducts influenced, and among them, the main factor which explains this bias is the higher diagnostic specificity (88.0%). Among the group of physicians who were not influenced, the major reason was not having access to this resource (55.6%). There was no statistical difference between the specialties of Neurology and Geriatrics in relation to the influence or not of biomarkers on their medical conduct (p = 0.058; Odds Ratio (OR) 1.54; Confidence Interval (CI) 0.98 - 2.41). Accordingly, the age groups 25-45 and 46-85 were not associated with the influence or not of biomarkers on the medical conduct among neurologists and geriatricians (p = 0.990; OR 0.99; CI 0.65 - 1.52).

Regarding the relationship between the presence of clinical symptoms of AD and biomarkers, in cases with positive biomarkers but without manifest symptoms, only 36.8% of all physicians would diagnose AD. Nevertheless, in the presence of specific clinical symptoms without biomarkers, 84.9% of all physicians would make the diagnosis. There was no statistical difference between the biomarkers and specialties, age group, gender or frequency in which AD diagnosis is revealed (p > 0.05).

## DISCUSSION

### Changes in the medical environment

Medical conduct towards AD diagnostic disclosure has changed over the years. In comparison to the study carried out by Raicher et al. 9 in 2008 also in Brazil, changes in the medical environment have been responsible for an increase in disclosure. In 16 years, the percentage of physicians who always or usually disclose the diagnosis has risen from 44.7% to 66.7%. On the other hand, the percentage of physicians who rarely or never do it has reduced from 25.4% to 12.3%, within the same period of time.

It is interesting to notice that younger specialists (aged 25 to 45 years) tend to disclose the diagnosis more often than older practitioners (aged 46 to 85 years), with a significant difference for neurologists from the two age groups. This feature is in agreement with the post-Flexner medical teaching models, which, as mentioned before, value patient autonomy, and most likely influenced the formation of younger physicians rather than the older ones. 8 Besides, for physicians working with AD for more than 10 years, 33.2% of them changed their behavior about diagnostic disclosure, now revealing the diagnosis, which is associated with a change in medical culture *per se* , not only among newly graduated generations.

Nevertheless, there still is, as noticed in 2008, a marked inconsistency between the physician's conduct towards pwAD and the wishes they would have if they would develop AD. The physicians consider that 74.8% of pwAD want to know their own diagnosis, meanwhile 96.2% of the physicians would want to know their own diagnosis. We have reasons to believe that the professionals recognize the importance of preparing for the future and dealing with issues associated with asset-management. Even so, they are discouraged to disclose the diagnosis due to several factors, such as the family members' opinion; pwAD desire to know the diagnosis; the remains of a paternalistic medical culture; among other motives.

Also in that matter, the use of precise terminology has not changed significantly in comparison to 2008: 84.7% of physicians use precise terminology to address the diagnosis, such as AD or dementia, in comparison to 85.2% in 2008. 9

### Mental health issues

There is still great concern with the pwAD mental health regarding diagnostic disclosure, especially in relation to the fear of destroying their hope and motivation, thus causing psychological distress. Furthermore, mental health issues are the main justification of physicians who did not change their approach and keep not disclosing the diagnosis. This was also seen in the 2008 study, 9 suggesting that this barrier has not yet been overcome.

It is known that dementia and depression are correlated. Depression is a risk factor for dementia, and it is under discussion if it is also a prodromal stage of AD. 10 Also, in pwAD, the onset of depression can exacerbate cognitive and functional impairment, reducing quality of life. 11 This can explain, although not justify, the decision of physicians not to disclose the diagnosis of dementia, considering the close relationship between cognitive and psychological functionality.

Besides, data shows that psychiatrists (75.9%) tend to disclose the diagnosis “always or usually” more frequently than neurologists (67.5%) and geriatricians (62.1%). Although the number of psychiatrists was much lower than the other two groups, these differences may be explained by the lack of medical training on mental health, seeing that psychiatrists are most likely better trained to deal with those concerns.

### The emergence of AD biomarkers

When considering the main characteristics that influence the decision to disclose or not the diagnosis, an important factor is the degree of diagnostic certainty. Concerning this subject, the detection of disease-specific biomarkers in cerebrospinal fluid (CSF) or with positron emission tomography contributes to a more accurate diagnosis of AD, even in early disease stages. 12

Accordingly, the degree of certainty of the diagnosis influences the decision to disclose or not the diagnosis, therefore making the emergence of biomarkers an important factor in favor of the disclosure. Despite the family members' opinions, the pwAD desire to know and the dementia stages are still important influencers on the decision. The positivity of biomarkers is the main declared reason for a change in physicians' behavior (24.1%) in favor of disclosing the diagnosis. When clinical symptoms are accompanied by positivity of biomarkers, diagnostic disclosure would occur in 84.9% of the cases.

Nevertheless, with the presence of biomarkers, but in the absence of clinical symptoms or when symptoms are nonspecific, only 36.8% of physicians are in favor of disclosure. In this sense, it should be questioned whether revealing the diagnosis would have any benefit in relation to the pwAD quality of life and mental health, because no specific treatments for these early stages are currently available.

At the moment, the Brazilian Academy of Neurology (BAN) indications for CSF examination are displayed on Table 4 . 13

**Table 4:** Describes the mainly indications for CSF examination by BAN

1. Investigation of presenile dementia (before 65 years)
2. Cases with atypical clinical presentation or course
3. Communicating hydrocephalus
4. Any evidence or suspicion of inflammatory, infectious, or prion disease of the central nervous system
5. If after the entire diagnostic process the etiology of the dementia syndrome remains doubtful

This study aimed to assess physicians' conduct towards AD diagnostic disclosure in Brazil, by also evaluating behavioral changes over time, drawing parallels with a similar study carried on in 2008. 9 It is interesting to notice changes in the medical environment during those 15 years, being the main noticeable result of the study, shown as the increase in revealing the diagnosis to the patients and their families. Mental health issues are still the main impeditive factor, counterbalanced by the arisal of biomarkers as a motivating factor for the disclosure.
