# Supplementary material for: Diagnostic disclosure of Alzheimer's disease in Brazil: a national survey of specialized physicians
Source: Arq Neuropsiquiatr. 2023 Nov 8;81(10):905–12. doi: 10.1055/s-0043-1776316 (PMC10631852; doi:10.1055/s-0043-1776316)
Supplement: Supplementary file 1 — Supplementary Material [file 10-1055-s-0043-1776316-s230114.pdf]

Supplementary Material 1 https://www.arquivosdeneuropsiquiatria.org/wp-content/uploads/2023/09/ANP-2023.0114-Supplementary-Material-1-e-2-.docx

Supplementary Material 2 https://www.arquivosdeneuropsiquiatria.org/wp-content/uploads/2023/09/ANP-2023.0114-Supplementary-Material-1-e-2-.docx

 © 2023. The Author(s).
